# Safety and efficacy of intratracheal DNase with physiotherapy in severe status asthmaticus

**DOI:** 10.1186/cc9605

**Published:** 2011-03-11

**Authors:** A Nyman, K Puppala, S Colthurst, S Parsons, S Tibby, I Murdoch, A Durward

**Affiliations:** 1 Evelina Childrens Hospital, Guy's and St Thomas' NHS Trust, London, UK

## Introduction

Diffuse airway plugging with thick viscous secretions is recognised in acute severe asthma, and contributes to airflow limitation in ventilated asthmaticus. Since 2004, we have used intratracheal DNase with physiotherapy as second-line therapy in mechanically ventilated children with severe status asthmatics who are refractory to conventional medical management. Our aim is to report the safety profile and efficacy of intratracheal DNase mucolytic therapy in this cohort.

## Methods

A retrospective cohort analysis in a 20-bed PICU. Forty-six ventilated children, median (IQR) age 74 months (45 to 141), received intratracheal DNase with physiotherapy (January 2004 to August 2010). Indication for DNase was peak inspiratory pressure (PIP) >28 cmH_2_O with hypercarbic acidosis (pCO_2 _> 10 kPa). Eleven patients required additional doses of DNase. In 40 episodes DNase was given blindly (*n *= 40) or bronchoscopically (*n *= 17).

## Results

**Figure 1:**
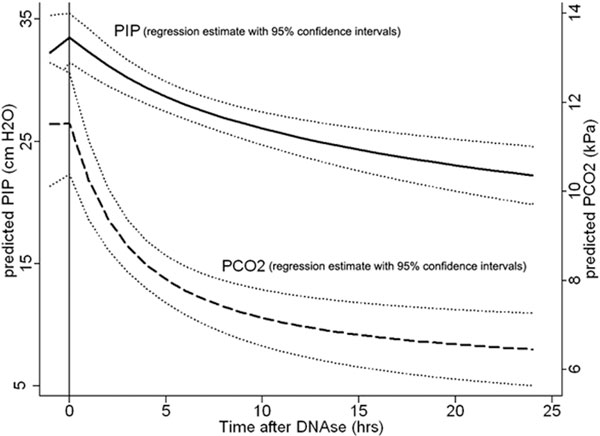
**Fractional polynomial regression of PIP/PCO_2 _following DNAse**.

The median (IQR) time to DNase following PICU admission was 2.1 hours (1.3 to 3.8). At the time of DNase, median PIP was 34 cm (30 to 40), pH was 7.12 (7.01 to 7.22) and pCO_2 _was 11 kPa (7.9 to 14.1). Overall DNase produced an improvement in ventilation (see Figure 1). Salbutamol IV was constant at 1 μg/kg/minute (0.5 to 2). The therapy was well tolerated with no hypoxic or hypotensive episodes, or air leaks. Median length of ventilation was 22 hours (15 to 37). No patient required extracorporeal membrane oxygenation and there were no deaths.

## Conclusions

Intratracheal DNase with physiotherapy is safe and effective therapy for refractory ventilated patients with status asthmatics. A randomised control trial is warranted.
